# Remote Patient Monitoring in Louisiana Medicare Beneficiaries With Diabetes or Hypertension: Retrospective Cohort Study of 2016-2020 Claims Data

**DOI:** 10.2196/80782

**Published:** 2026-03-03

**Authors:** Debra Winberg, Charles Stoecker, Jian Li, Lizheng Shi

**Affiliations:** 1 Department of Health Management and Policy, School of Health, Georgetown University, St Mary's Hall, 3700 Reservoir Road, Washington, DC, 20057, United States, 1 202-687-4221; 2 Department of Health Policy and Management, Celia Scott Weatherhead School of Public Health and Tropical Medicine, Tulane University, New Orleans, LA, 20057, United States; 3 Department of Biostatistics, Celia Scott Weatherhead School of Public Health and Tropical Medicine, Tulane University, New Orleans, LA, 20057, United States

**Keywords:** remote patient monitoring, diabetes, hypertension, health utilization, medicare

## Abstract

**Background:**

Chronic conditions such as diabetes and hypertension pose major public health and economic challenges. Innovative tools, including remote patient monitoring (RPM), aim to address barriers to care by digitally transmitting health data to providers.

**Objective:**

This study assesses the impact of RPM on health care utilization in Medicare beneficiaries with diabetes or hypertension.

**Methods:**

This retrospective cohort study used a longitudinal panel from 2016 to 2020 based on Louisiana Medicare Fee-for-Service claims and Beneficiary Files. Patients were included if they were diagnosed with diabetes or hypertension before 2016. RPM exposure was defined by common procedure terminology/healthcare common procedure coding system codes (eg, 99453, 99454, 99457, 99458, 95250, 95251, 99091, E2100-E2103, E0607), with the corresponding month considered the treatment month. Control beneficiaries were randomly assigned treatment months matching the treatment group. Outcomes were monthly counts per 1000 beneficiaries of outpatient, emergency department, and inpatient visits (all-cause and disease-specific), constructed via revenue center/place-of-service/primary ICD-10 (International Classification of Diseases-10) codes. We estimated inverse-probability-treatment-weighted 2-way fixed-effects models with standard errors clustered at the physician level. Data represent secondary analysis of deidentified claims; no participant recruitment occurred. All confidence intervals are reported at the 95% confidence level.

**Results:**

The study included 5488 beneficiaries in the treatment group and 341,226 in the control group. RPM was associated with increased level of outpatient visits in the treatment group by 2284.01 (95% CI 2059.75-2508.27) visits per month per 1000 people (*P*<.001) after initiation and decreased the trend of outpatient visits by 37.61 (95% CI −37.98 to −37.24) visits per month per 1000 people compared with the control group (*P*<.001). Similarly, the level of emergency room visits increased in the treatment compared with the control by 84.60 (95% CI 72.21 to 96.99) visits per month per 1000 people initially after RPM initiation (*P*<.001) and decreased the trend by 1.40 (95% CI −1.60 to −1.20) visits per month per 1000 people (*P*<.001). The level of inpatient visits increased by 32.64 (95% CI 24.84-40.44) visits per month per 1000 beneficiaries in the treatment group compared with the control immediately after RPM prescription (*P*=.002), but the trend decreased by 0.54 (95% CI −0.66 to −0.42) visits per 1000 people each month (*P*<.001).

**Conclusions:**

This study is among the first large, claims-based evaluations of RPM in older adults with diabetes or hypertension using a beneficiary- and time-fixed-effects design. We identify a pattern of increased health care utilization following RPM initiation, followed by a gradual decline that does not fully offset the initial increase. By characterizing utilization trajectories and not clinical endpoints, this study helps reconcile inconsistent findings in prior RPM research and highlights the importance of implementation factors in Medicare populations. Strategies to maximize the value of RPM may include appropriate population targeting, patient education on digital health technologies, and clinician workflow support to support effective integration into routine care.

## Introduction

Chronic conditions, including diabetes and hypertension, are significant public health challenges. In the past 30 years, the prevalence of diabetes has increased by 11%, and in 2024, more than 30 million Americans were diagnosed with diabetes [1]. Similarly, hypertension affects approximately 47% of Americans, or over 120 million people [2,3]. Both diseases are major contributors to other chronic health concerns, including cardiovascular disease, chronic kidney disease, stroke, and premature mortality, which can also increase rates of health care resource utilization (HCRU). More so, the economic impact is significant. Both diabetes and hypertension are leading causes of morbidity and mortality in the United States, with combined mortality rates nearly doubling from 2000 to 2023 [4]. In 2022, hypertension-related costs exceeded US $219 billion annually, and diabetes direct costs were over US $300 billion [5-9].

This burden disproportionately impacts older adults over 65, who face higher rates of both conditions in addition to higher rates of complications, multimorbidity, and polypharmacy [7,10]. In turn, Medicare bears much of the burden from these conditions [7].

There are many successful pharmacological treatments and evidence-based lifestyle interventions for diabetes and hypertension, yet over half of patients do not have their blood sugar or blood pressure under control [11,12]. Alarmingly, control rates have worsened in recent years, with hypertension control decreasing from 54% in 2013 to 48% in 2017‐2020 and continuing to decrease during the COVID-19 pandemic [13,14]. Similarly, glycemic control has stagnated in the past 30 years, and disparities in control have increased [15]. These trends highlight persistent barriers in chronic disease management, including poor medication adherence, limited access to specialty care, high costs, social and economic challenges, and low patient engagement in care [16]. Innovative digital tools, including remote patient monitoring (RPM), aim to address these barriers and improve disease control. RPM is an mHealth device that aims to improve patient care by digitally transmitting health data from patients to physicians [17]

By reducing patient travel costs, allowing physicians to treat more patients, facilitating practice management, and providing health care providers access to patient data quickly, these devices target several barriers to glycemic control, including nonadherence, self-management, and health care experiences [18-20]. RPM adoption in the United States has accelerated in recent years, driven by expanded reimbursement policies, technological advances in wearables and connected devices, and the growing need to manage chronic disease more effectively [21]. These factors have positioned RPM as one of the most commonly used digital health tools, particularly for conditions such as diabetes and hypertension.

Evidence regarding RPM’s effectiveness is positive, but inconsistent. A 2024 systematic review on RPM interventions found measurable impacts in chronic disease outcomes, though effects varied based on a number of variables, including the specific disease and implementation design [22]. A review of systematic reviews found that there are possible benefits from RPM, but results are inconsistent [23]. For example, several trials and meta-analyses report A1c reductions by 0.3%‐1.22% and decreases in systolic blood pressure of around 4‐9 mmHg among RPM cohorts [17,24-26]. While these are modest changes, they can be clinically meaningful and suggest RPM can support better chronic disease control.

However, not all evidence is positive; some studies demonstrate that the RPM has no impact and may only be effective for certain patient groups [27-33]. Specifically, RPM may only benefit specific subgroups, such as people with a high baseline risk, who monitor very often, or who are very engaged in their care and have physicians who are very engaged in their care [34]. Additionally, there is mixed evidence on the ability of RPM to decrease HCRU [35,36]. Some studies found that RPM can prevent costly acute events and reduce hospitalizations and emergency room (ER) visits and may be cost-effective for hypertensive patients [37]. However, other studies demonstrated no change or increased utilization patterns, potentially attributable to the detection of previously unaddressed clinical needs or enhanced surveillance following RPM deployment [38]. Overall, the effectiveness of RPM and the balance between increased monitoring and reduced acute care use remain unclear.

Despite the rapid expansion of RPM coverage and use, most of the existing literature focuses on younger and commercially insured populations who may not have such a high burden of diabetes and hypertension and have fewer barriers to using RPM**,** which may impact its effectiveness [39,40]. While the evidence base in Medicare beneficiaries exists, it remains limited and often focuses on other conditions such as heart disease and chronic obstructive pulmonary disease (COPD) [35,41]. A recent systematic review found that most of the studies on RPM focus on usability and feasibility, not outcome measures such as utilization, for cardiovascular disease [42]. Therefore, not enough real-world evidence studies have been performed for diabetes and hypertension. As most general RPM services in Medicare beneficiaries are for diabetes and hypertension, understanding their impact on this population is essential. Additionally, as some of the Medicare literature shows a more negative impact, such as increased health utilization, it is essential to analyze more specific Medicare populations [38].

This study aims to assess the impact of RPM on HCRU in Medicare beneficiaries with diabetes or hypertension. By focusing on an older, higher-risk population, our analysis contributes novel real-world evidence on the effectiveness of RPM and provides timely insights for policymakers and providers considering broader adoption.

## Methods

### Overview

This is a retrospective cohort study using Louisiana Medicare Fee-for-Service claims linked to beneficiary files and analyzed using an inverse probability treatment weighted (IPTW) controlled interrupted time series (CITSA) design. This manuscript was prepared following the Strengthening the Reporting of Observational Studies in Epidemiology (STROBE) reporting guidelines [43].

### Data Source and Patient Selection

We used data from a patient-level longitudinal panel from January 1, 2016, to December 31, 2020, which was constructed from Louisiana Medicare Fee-for-Service claims and Beneficiary files. This data is collected at the time of service and was purchased from the Research Assistance Data Center for secondary analyses. We included patients who had a diagnosis of diabetes or hypertension based on the CMS Chronic Conditions files. Patients were excluded if they did not have a diabetes or hypertension diagnosis before 2016, were not enrolled in Medicare before 2016, had only gestational diabetes or secondary diabetes, or had end-stage renal disease. These exclusions were found using the ICD codes.

Patients were included in the treatment group if they had at least one occurrence of a common procedure terminology (CPT) code for RPM or continuous glucose monitoring (99453, 99454, 99457, 99458, 95250, 95251, 99091, E2100, E0607, E2102, E2103, E2101). This information was found in the inpatient, ER, or outpatient claims files. We defined the treatment month as the first month with this CPT code. For the control group, pseudo-treatment dates were randomly assigned to reflect the distribution of treatment initiation in the intervention group. Specifically, in Excel, each month and year combination was coded numerically, and controls were assigned a treatment month and year in proportion to the observed distribution of initiation dates in the treatment group. This ensured alignment by time of year rather than only by year, reducing bias from differential calendar-time exposure. After the assignment, we conducted balance checks and confirmed that the month-by-month distribution of treatment dates did not significantly differ between the control and treatment groups.

### Outcome Variables

The number of outpatient, ER, or inpatient visits per month. We defined these outcomes using published guidance from the Research Data Assistance Center using revenue center codes, claim dates, and places of service codes in the Medicare Fee-for-Service files (Carrier claims, Carrier Line, Base Claims, and Revenue Center). The disease-specific outcomes included the number of outpatient, ER, or inpatient visits per month where the primary ICD code indicated that the visit was for diabetes or hypertension. If a beneficiary did not have an encounter in a certain month, they were coded as having 0 visits. Visits with RPM as the primary CPT code were not included. All outcomes were detrended to remove underlying time trends to focus on deviations that may be associated with RPM.

### Statistical Analysis

We used a CITSA to determine the impact of RPM. This approach leverages pre- and posttreatment data from a treatment and control group to estimate changes in the level and slope of the outcome variables before and after treatment initiation. This method assesses the impact of an intervention such as RPM by comparing a treatment to a control group over time. CITSA was chosen for the analysis because of the linearity of the data over time (Multimedia Appendix 1) and for its ability to account for pre-existing trends and provide a counterfactual estimate. The CITSA model may be written as:


$$Y=\beta_{o}+\beta_{1}\left(Time_{t}\right)+\beta_{2}\left(RPM_{i}\right)+\beta_{3}\left(time_{t}*RPM_{i}\right)+\beta_{4}\left(post_{t}*RPM_{i}\right)+\beta_{5}\left(time_{t}*RPM_{i}*post_{t}\right)+\beta_{6}X+\epsilon$$

The baseline level is given by the intercept β_0_. β_1_ represents the underlying time trend for the non-RPM group before the intervention. The indicator variable RPM specifies group membership, with β_2_ capturing any baseline differences between the intervention and control groups. The term β3 allows for differences in preintervention trends between RPM and non-RPM groups. The coefficient β_4_ represents the immediate postintervention effect. The 3-way interaction term β_5_ captures any differential trend change attributable to the intervention over time. There was no specific postperiod variable included in the model, as the control group had a randomly assigned treatment date and, as such, had no expected postperiod outcome change.

To account for bias and confounding, we adjusted for covariates that may influence the outcome, including comorbidities (diabetes, hypertension, COPD, chronic kidney disease, hyperlipidemia), Medicare status, sex, race (White, Black, Hispanic, Asian, Other), rurality (defined as metropolitan, micropolitan, rural using FIPS codes and 2020 core-based statistical area data), and age. These variables were created using the Medicare Beneficiary Files (chronic condition and ABCD files), which aggregate data at the annual level.

All analyses accounted for time- and beneficiary-level fixed effects and were weighted by the IPTW, and standard errors were clustered at the primary doctor level to account for serial correlations over time. To construct the weights, we modeled the probability of RPM participation using baseline demographic and clinical characteristics from the month before treatment and the average preperiod outcomes. Each beneficiary was assigned a weight equal to the inverse of their predicted probability. This data was obtained from the beneficiary files and the previously created outcome variables. The primary doctor was defined as the physician whom a beneficiary saw the most during the study period to account for correlated practice patterns. In the base case, the main model used all available data in the 60 months regardless of pre- and postlength of time.

We used an inverse-probability–weighted 2-way fixed-effects model to control for unobserved, time-invariant differences across beneficiaries and shared temporal shocks. This design isolates within-beneficiary changes before and after RPM initiation, consistent with the interrupted time series framework. It was preferred to multilevel models because exposure timing varies across beneficiaries, and fixed effects more clearly estimate pre/post level and trend differences [44].

Because Medicare claims are used for reimbursement, demographic, enrollment, and utilization fields are complete for almost all beneficiaries. Diagnosis and procedure variables used to construct comorbidities are also not subject to item-level analysis. Multiple imputation was not performed, as missingness was minimal and largely structural and less than 15% for all key variables.

All confidence intervals were calculated at the 95% confidence level. We tested the robustness of the results by clustering standard errors by zip code and changing the necessary length of pre- and posttreatment data to 6 months pre and 6 months post, and 12 months pre and 12 months post. Additionally, we assess seasonality of treatment by running the analysis with a variable for calendar month (January, February, etc) and another analysis with a control variable for calendar month of treatment. Additionally, we ran an analysis excluding COVID years to ensure we were not picking up COVID confounding and to account for potential exposure classification issues due to new CPT codes being introduced.

All analyses were conducted using Stata (version 18.0; IBM Corp).

### Ethical Considerations

This study was approved by Tulane University’s Institutional Review Board (2023-1044-SPHTM) through an expedited review, as it only uses secondary data. The data use agreement and institutional review board for this study allow the use of secondary data analysis without additional consent from beneficiaries. All study data is deidentified to protect beneficiaries, and there was no way to reidentify. As we did not work directly with beneficiaries, no compensation was given, and there is no identification of individuals in this manuscript.

## Results

Initially, the data included 480,592 Louisiana beneficiaries with diabetes or hypertension. This sample included 472,902 beneficiaries in the control group. Of these, 118,513 were excluded for being diagnosed with hypertension or diabetes after 2016. A further 9 beneficiaries were dropped for being enrolled in Medicare too late, and 130 were excluded for only having secondary diabetes. Last, 20,226 beneficiaries were dropped for having end-stage renal disease.

Initially, 7690 Medicare beneficiaries were identified in the treatment group. Of these, 1849 beneficiaries were excluded because they were diagnosed with hypertension or diabetes after 2016. No beneficiaries were excluded due to delayed Medicare enrollment, 2 beneficiaries were excluded for having secondary diabetes only, and 351 beneficiaries were excluded due to end-stage renal disease. After applying all inclusion and exclusion criteria, a total of 346,714 Medicare beneficiaries with diabetes or hypertension were included in the analytic sample. The final sample comprised 5488 beneficiaries in the treatment group and 341,226 in the control group. Among the treated beneficiaries, 719 (13.1%) initiated RPM in 2016, 635 (11.6%) in 2017, 770 (14.0%) in 2018, 1346 (24.5%) in 2019, and 2018 (36.8%) in 2020. These distributions remained balanced after weighting.

Table 1 presents the weighted and unweighted baseline characteristics of the treatment and control groups. After weighting, all standardized mean differences were below 10%. Prior to weighting, the treatment group had a higher prevalence of several chronic conditions, including diabetes (3842, 70% vs 112,605, 33%), hypertension (5049, 92% vs 245,683, 72%), COPD (494, 9% vs 23,886, 7%), chronic heart failure (823, 15% vs 30,710, 9%), chronic kidney disease (2031, 37% vs 47,772, 14%), and hyperlipidemia (2524, 46% vs 88,719, 26%).

The RPM group was younger on average (mean age 71.6 vs 74.2 years), included a higher proportion of male beneficiaries (2470, 45% vs 126,254, 37%), and a higher proportion of White beneficiaries (3842, 70% vs 208,148, 61%). In contrast, beneficiaries in the treatment group were less likely to have dual Medicaid eligibility (1317, 24% vs 139,903, 41%). The treatment group also demonstrated higher baseline health care utilization, including emergency department visits, inpatient diabetes-related visits, outpatient visits, and outpatient diabetes-related visits. After weighting, baseline demographic characteristics, clinical conditions, and health care utilization were comparable between the treatment and control groups.

**Table 1.** Baseline population characteristics and health care utilization. Baseline demographic, clinical, and health care utilization characteristics of Medicare Fee-for-Service beneficiaries in Louisiana with hypertension or diabetes, 2016-2020. Patients in the treatment group initiated remote patient monitoring or continuous glucose monitoring based on relevant procedure codes. Data are unweighted and weighted using inverse probability treatment weight, which were created using demographic and utilization data. Values are shown as means (SD) or percentages, with standardized mean differences (SMD) presented for both unweighted and weighted comparisons. Utilization outcomes are average monthly counts in the preintervention period.

Variable	Nonweighted			Weighted		
	Treatment mean (SD) (n=5488)	Control mean (SD) (n=341,226)	SMD	Treatment mean (SD) (n=5488)	Control mean (SD) (n=341,226)	SMD
Diabetes	0.70 (0.46)	0.46 (0.47)	−0.81	0.70 (0.46)	0.71 (0.45)	0.02
Hypertension	0.92 (0.27)	0.72 (0.45)	−0.54	0.92 (0.27)	0.93 (0.26)	0.02
Chronic obstructive pulmonary disease	0.09 (0.29)	0.07 (0.25)	−0.1	0.09 (0.29)	0.10 (0.30)	0.01
Heart failure	0.15 (0.36)	0.09 (0.29)	−0.19	0.15 (0.36)	0.16 (0.36)	0.01
Chronic kidney disease	0.37(0.48)	0.14 (0.35)	−0.54	0.37 (0.48)	0.36 (0.48)	−0.01
Hyperlipidemia	0.46 (0.50)	0.26 (0.44)	−0.44	0.46 (0.50)	0.46 (0.50)	−0.01
Myocardial infarction	0.01 (0.11)	0.00 (0.07)	−0.08	0.01 (0.11)	0.01 (0.11)	0
Charlson Comorbidity Index	3.88 (2.52)	2.46 (2.50)	−0.56	3.88 (2.52)	4.10 (3.15)	0.08
Age	71.58 (10.36)	74.15 (11.27)	0.24	71.58 (10.36)	71.72 (10.56)	0.01
Dual status	0.24 (0.43)	0.41 (0.49)	0.36	0.24 (0.43)	0.27 (0.44)	0.08
Percent male	0.45 (0.50)	0.37 (0.48)	−0.17	0.45 (0.50)	0.44 (0.50)	−0.02
Percent White	0.70 (0.46)	0.61 (0.49)	−0.19	0.70 (0.46)	0.70 (0.46)	0.00
Percent Black	0.26 (0.44)	0.23 (0.42)	−0.07	0.26 (0.44)	0.26 (0.44)	0.00
Percent Hispanic	0.02 (0.14)	0.02 (0.13)	−0.03	0.02 (0.14)	0.02 (0.14)	−0.01
Percent Asian	0.01 (0.09)	0.01 (0.08)	−0.02	0.01 (0.09)	0.01 (0.09)	−0.01
Percent metropolitan	0.83 (0.38)	0.75 (0.43)	−0.19	0.83 (0.38)	0.84 (0.37)	−0.01
Percent micropolitan	0.09 (0.29)	0.14 (0.35)	0.15	0.09 (0.29)	0.09 (0.28)	−0.01
Percent rural	0.08 (0.27)	0.11 (0.31)	0.11	0.08 (0.27)	0.08 (0.27)	−0.01
Inpatient visits	0.03 (0.06)	0.03 (0.06)	−0.04	0.03 (0.06)	0.03 (0.07)	0.09
Inpatient visits (hypertension)	0.00 (0.01)	0.00 (0.01)	−0.03	0.00 (0.01)	0.00 (0.01)	0.02
Inpatient visits (diabetes)	0.00 (0.02)	0.00 (0.01)	−0.13	0.00 (0.02)	0.00 (0.01)	−0.05
Emergency room visits	0.01 (0.03)	0.00 (0.01)	−0.22	0.01 (0.03)	0.00 (0.03)	−0.08
Emergency room visits (diabetes)	0.00 (0.02)	0.00 (0.01)	−0.06	0.00 (0.02)	0.00 (0.02)	−0.01
Emergency room visits (hypertension)	0.07 (0.10)	0.06 (0.09)	−0.12	0.07 (0.10)	0.08 (0.11)	0.06
Outpatient visits	2.34 (1.60)	1.53 (1.52)	−0.52	2.34 (1.60)	2.27 (1.92)	−0.04
Outpatient visits (diabetes)	0.34 (0.41)	0.08 (0.20)	−0.83	0.34 (0.41)	0.29 (0.60)	−0.10
Outpatient visits (hypertension)	0.14 (0.17)	0.12 (0.21)	−0.08	0.14 (0.17)	0.14 (0.21)	0.01

**Figure 1.**
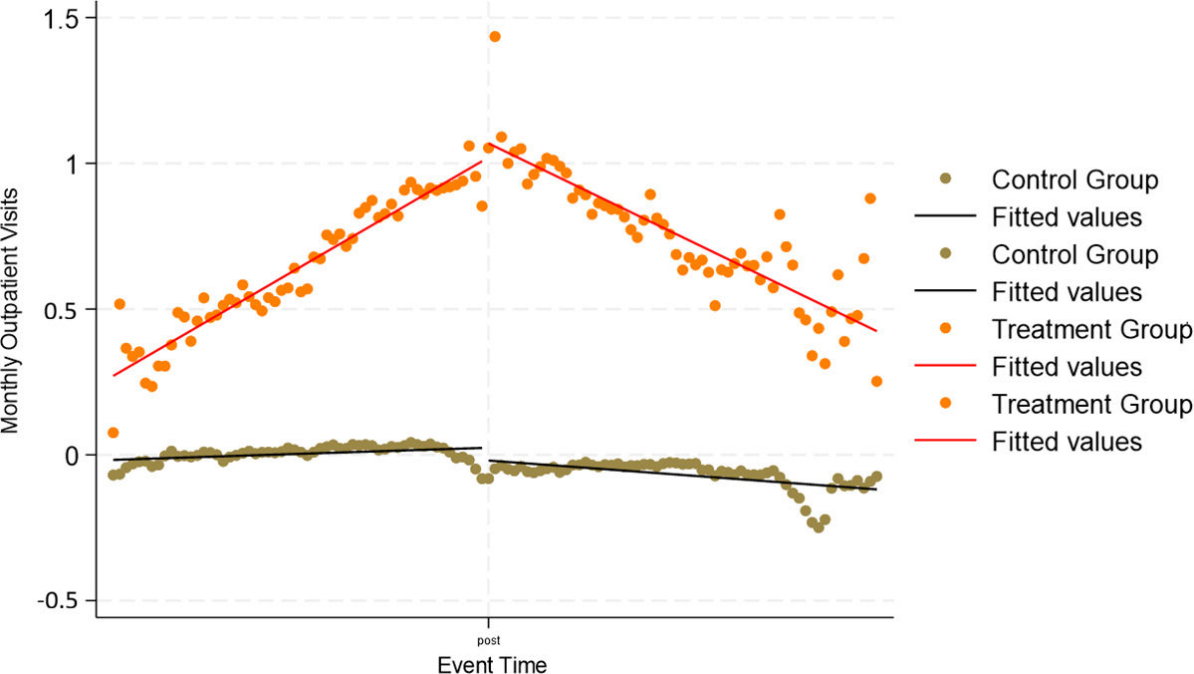
Residualized monthly outpatient visits before and after remote patient monitoring initiation. Models were adjusted for age, sex, race, rurality, comorbidities (diabetes, hypertension, chronic obstructive pulmonary disease, chronic kidney disease, hyperlipidemia), and Medicare status.

**Figure 2.**
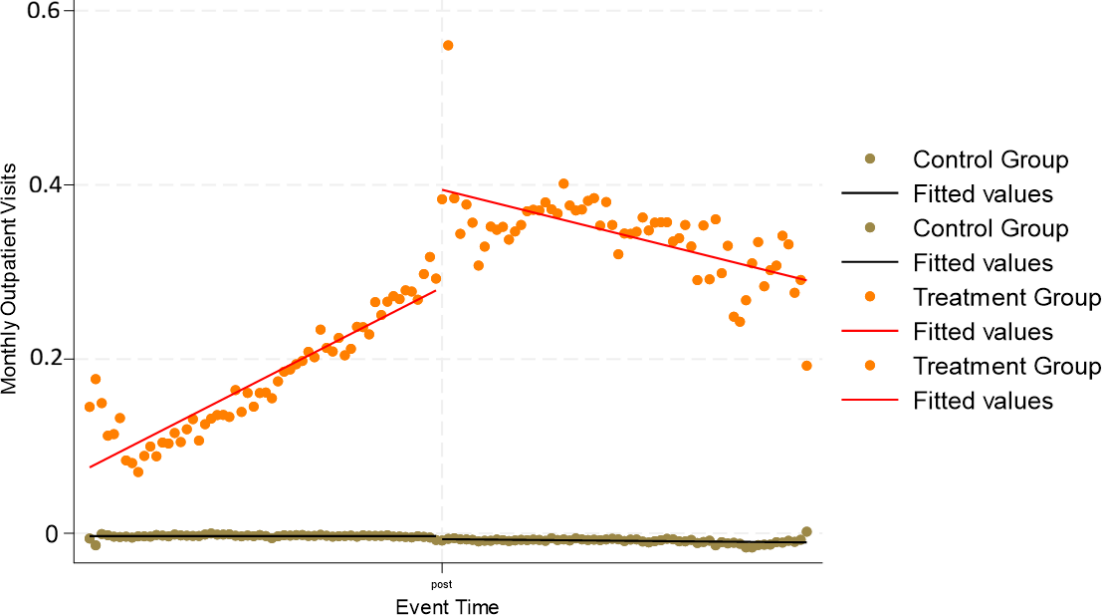
Residualized monthly emergency room visits before and after remote patient monitoring initiation. Models were adjusted for age, sex, race, rurality, comorbidities (diabetes, hypertension, chronic obstructive pulmonary disease, chronic kidney disease, hyperlipidemia), and Medicare status.

**Figure 3.**
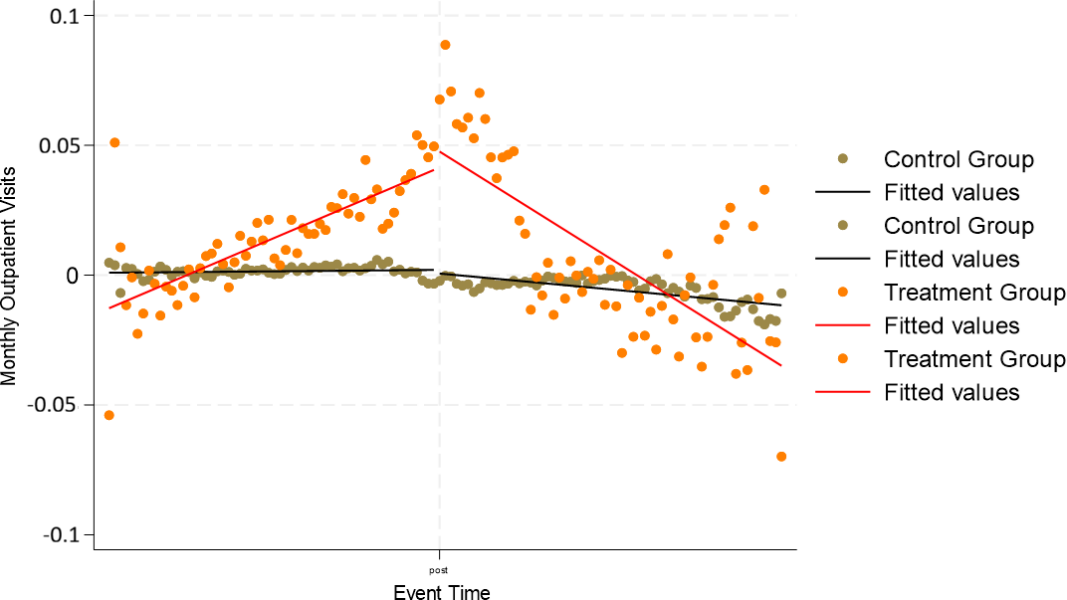
Residualized monthly inpatient visits before and after remote patient monitoring initiation. Models were adjusted for age, sex, race, rurality, comorbidities (diabetes, hypertension, chronic obstructive pulmonary disease, chronic kidney disease, hyperlipidemia), and Medicare status.

Figures 1-3 display residualized monthly outpatient, ER, and inpatient visits by treatment status. These figures plot model residuals after covariate adjustment and illustrate underlying utilization patterns. For outpatient visits (Figure 1), the treatment group increased from about 0.4 residual visits per month preintervention to a peak near 1.2 around the index date, before declining to approximately 0.5 by the end of follow-up. The control group residuals hovered close to 0 throughout. For ER visits (Figure 2), the treatment group rose to roughly 0.035 residual visits per month at the peak, declining back toward zero postintervention. In contrast, the control group stayed flat at approximately 0 residual visits. For inpatient visits (Figure 3), the treatment group showed a smaller preintervention increase, reaching about 0.018 residual visits per month, followed by a decline toward baseline. Again, the control group remained stable near 0 across the entire period. Residual plots for disease-specific outcomes can be found in Multimedia Appendix 1.

Table 2 depicts the CITSA results. All-cause outpatient visits steadily increased by 9.81 (95% CI 2.42-17.20; *P*=.01) visits per month per 1000 people during the time period. In the preperiod, beneficiaries in the treatment group had their visits decrease by 10.83 (95% CI −18.45 to 3.21; *P*=.01) visits per month per 1000 people due to treatment. After RPM initiation, the level of outpatient visits increased by 2284.01 (95% CI 2059.75-2508.27) visits per month per 1000 people (*P*<.001), and the trend in the number of outpatient visits decreased by 37.61 (95% CI −37.98to −37.24) visits per month per 1000 people compared with those in the control group (*P*<.001). Diabetes-specific visits did not change significantly over the time period or in the pretreatment time period. The level of diabetes-specific appointments increased by 673.26 (95% CI 586.98-759.54) visits per month per 1000 people (*P*<.001) after initiation and then decreased at a higher rate of 10.25 (95% CI −11.39 to −9.11) visits per month per 1000 people (*P*<.001). Over the entire study period, hypertension outpatient visits increased by 0.47 (95% CI 0.31 to 0.63) visits per month per 1000 people (*P*<.001), but did not significantly change in the pretreatment period. The level of hypertension visits increased by 190.92 (95% CI 136.06-245.78) visits in the RPM group compared with those in the control group (*P*<.001), while the trend decreased by 2.91 (95% CI −3.58 to −2.2) visits per 1000 people over the next several years.

All-cause ER visits increased by 0.27 (95% CI 0.17-0.37) visits per month per 1000 people over the entire study period (*P*<.001). In the pretreatment period, visits decreased by 0.3 (95% CI −0.44 to −0.16) visits per month per 1000 people (*P*<.001). Compared with the control, the level of number of ER visits increased by 84.60 (95% CI 72.21-96.99) visits per 1000 people initially after RPM initiation (*P*<.001) and the trend decreased by 1.40 (95% CI −1.60 to −1.20) visits per month per 1000 people (*P*<.001). While the overall time trend and pretreatment trend are insignificant, the patterns also hold for disease-specific ER visits after RPM initiation. At the time of RPM, the level of ER visits increased by 14.58 (95% CI 10.70-18.46) visits per month per 1000 beneficiaries and had a decreasing trend with 0.26 (95% CI −0.32 to −0.20) fewer visits per month per 1000 people (*P*<.001 for all outcomes) in the treatment compared with the control. Like diabetes-specific visits, there were no statistically significant overall time or pretreatment trends. The level of hypertension-specific visits increased by 5.68 (95% CI 2.56-8.80) visits per month for 1000 people after RPM initiation in the treatment group, compared with the control, and the trend decreased by 0.09 (95% CI −0.13 to −0.05) visits per month per 1000 people (*P*<.001) in the RPM group compared with the control.

**Table 2.** Impact of the RPM on health care resource utilization. All estimates are expressed as monthly visits per 1000 beneficiaries. Diabetes and hypertension-specific visits are based on the primary diagnosis code associated with the visit. Each cell contains the treatment effect and confidence intervals. Impact of remote patient monitoring (RPM) on monthly outpatient, emergency room, and inpatient visits among Medicare beneficiaries with hypertension or diabetes, Louisiana, 2016-2020. Estimates from inverse probability treatment weight-controlled interrupted time series models are shown with 95% confidence intervals and *P* values.

Variables	All-cause visits		Diabetes visits		Hypertension visits	
	OR^a^ (95% CI)	*P* value	OR (95% CI)	*P* value	OR (95% CI)	*P* value
Outpatient visits						
Time trend	9.81 (2.42 to 17.20)	.01	5.89 (−1.05 to 12.83)	.10	0.47 (0.31 to 0.63)	<.001
Pretreatment trend	−10.83 (−18.45 to 3.21)	.01	−6.23 (−13.19 to 0.73)	.08	−0.28 (−0.79 to 0.23)	.22
Posttreatment level	2284.01 (2059.75 to 2508.27)	<.001	673.26 (586.98 to 759.54)	<.001	190.92 (136.06 to 245.78)	<.001
Posttreatment trend	−37.61 (−37.98 to −37.24)	<.001	−10.25 (−11.39 to −9.11)	<.001	−2.91 (−3.58 to −2.24)	<.001
Emergency room visits						
Time trend	0.27 (0.17 to 0.37)	<.001	0.00 (0.00 to 0.00)	.48	0.00 (−0.02 to 0.02)	.62
Pretreatment trend	−0.30 (−0.44 to −0.16)	<.001	−0.01 (−0.03 to 0.01)	.66	0.00 (−0.02 to 0.02)	.87
Posttreatment level	84.60 (72.21 to 96.99)	<.001	14.58 (10.70 to 18.46)	<.01	5.68 (2.56 to 8.80)	<.001
Posttreatment trend	−1.40 (−1.60 to −1.20)	<.001	−0.26 (−0.32 to −0.20)	<.001	−0.09 (−0.13 to −0.05)	<.001
Inpatient visits						
Time trend	0.21 (0.17 to 0.25)	<.001	0.01 (0.01 to 0.01)	<.01	0.00 (−0.02 to 0.02)	.86
Pretreatment trend	−0.23 (−0.31 to −0.15)	<.001	−0.01 (−0.03 to 0.01)	.23	−0.01 (−0.03 to 0.01)	.48
Posttreatment level	32.64 (24.84 to 40.44)	<.001	5.08 (2.67 to 7.49)	<.001	2.82 (0.43 to 5.21)	.02
Posttreatment trend	−0.54 (−0.66 to −0.42)	<.001	−0.10 (−0.32 to −0.20)	<.001	−0.04 (−0.08 to −0.00)	<.001
Observations	20,799,900	—^b^	20,799,900	—	20,799,900	—

a OR: odds ratio.

b Not applicable.

All-cause inpatient visits increased by 0.21 (95% CI 0.17-0.25) visits per month per 1000 people over the entire study period (*P*<.001). In the pretreatment period, the trend decreased by −0.23 (95% CI −0.31 to −0.15) visits per month per 1000 people (*P*<.001). Compared with the control group, the level of all-cause inpatient visits increased by 32.64 (95% CI 24.84-40.44) visits per month per 1000 people in the treatment group compared with the control group immediately after RPM prescription (*P*<.001). The trend decreased by 0.54 (95% CI −0.66 to −0.42) visits per 1000 people each month (*P*<.001). During the study period, inpatient diabetes visits increased by 0.01 (95% CI 0.01-0.01) visits per month per 1000 people (*P*<.001) but were not statistically significant in the preperiod. The level of inpatient visits with diabetes as the primary diagnosis code increased by 5.08 (95% CI 2.67-7.49) visits per month per 1000 people right after treatment (*P*<.001), and the trend decreased by 0.01 (95% CI 2.51-2.31) visits per month per 1000 people (*P*<.001) in the RPM group compared with the control. Hypertension-specific inpatient visit levels increased 2.83 (95% CI 0.43-5.21) visits per month per 1000 people immediately after treatment (*P*=.02), and the trend decreased by 0.04 (95% CI −0.08 to 0.00) visits per month per 1000 (*P*<.001).

The results were robust to cluster changes and excluding COVID-19 months (Multimedia Appendix 2). Additionally, when assessing seasonality, both analyses remained statistically significant with the same overall patterns; however, the point estimates slightly decreased (Multimedia Appendix 2), showing there may be some seasonality. The results were not robust to changing the study time (limited to 6 or 12 mo).

## Discussion

### Principal Findings

This study aimed to assess the impact of RPM on HCRU on Louisiana Medicare beneficiaries with diabetes or hypertension from 2016 to 2020. By combining a rigorous longitudinal design with a focus on an older, high-burden Medicare population, this study provides novel insights into how RPM adoption affects health care utilization in real-world settings. Overall, in beneficiaries with diabetes or hypertension, RPM was associated with a statistically significant increase in outpatient, ER, and inpatient visits immediately after initiation, followed by a significant downward trend over time. These findings, supported by narrow confidence intervals, suggest that the estimates are precise and due to RPM, not random variation.

Existing evidence on RPM’s impact on utilization is mixed. Some studies report decreases in HCRU [35,45]. Others have shown that it may not change utilization [17]. Notably, studies in a Medicare population also demonstrated an increase in utilization; for example, one study on Medicare hypertensive patients reported an increase in cardiovascular outpatient visits [38]. Differences across studies may reflect variation in patient populations, RPM modalities, and social determinants of health. For example, our Medicare population may have lower digital or health literacy.

Additionally, the average beneficiary in our cohort was on RPM for 7 months, and results were not robust to a short time period, which is consistent with literature that found that clinical outcomes, such as decreased blood glucose, which may impact utilization, are only found in longer time periods [46]. Thus, long-term users may be driving the observed effects, underscoring the importance of sustained participation for realizing RPM’s full impact.

The dynamic trend of early increases followed by subsequent declines in utilization aligns with the literature, suggesting that individuals initially engage more intensively with new tools before stabilizing into routine patterns [47,48]. In the initial phase of RPM adoption, both patients and providers may be adjusting to new data and workflows. Patients may respond to frequent health alerts or health data with heightened awareness, prompting additional interactions with the health system. Providers may proactively schedule follow-ups to verify readings, resolve technical issues, or re-evaluate treatment plans due to the additional data.

The subsequent decrease might indicate that as patients become more accustomed to the data, they take a more measured approach to seeking care. It remains unclear whether these patterns represent appropriate, preventive care or unnecessary and wasteful utilization. The increase could stem from useful data and engagement that identify unmet needs and ensure patients are getting necessary care, or it could represent low-value encounters due to the large influx of data or cautious clinical behavior. Without information on clinical appropriateness, clinical outcomes, and more information on the visits, it is not possible to determine whether the short-term increase benefits patients and the health system.

This observed pattern likely reflects a complex interaction of patient and provider behaviors shaped by differences in digital literacy, clinical workflows, and familiarity with RPM. For instance, improved patient self-management is one of the key benefits of RPM and can lead to better outcomes, but it is dependent on patients’ ability to understand health data [49,50]. Both health and digital literacy are associated with age and education [50]. Given that this study focused primarily on older adults in a state with relatively low average education levels, some patients may have lacked the digital or health literacy required to meaningfully engage with RPM. This could limit the intervention’s potential benefits or even contribute to uncertainty-driven care-seeking rather than reducing visits.

Additionally, our study focused on early RPM adopters and physicians who were still learning effective RPM implementation methods. Clinicians were still learning how to interpret continuous data, integrate RPM into workflows, and communicate with patients. A systematic review of factors of RPM success highlights several physician-level factors for effectiveness, including targeting populations at high risk, providing responsive and timely care, and ensuring collaborative and coordinated care [45]. If providers are learning how to provide timely and coordinated care via new technologies, RPM may not have its intended benefit. Together, these patient- and provider-level factors suggest that the short-term rise in utilization may reflect a broader adaptation period as both groups learned to navigate new technologies and patterns of care.

Moreover, this study focuses specifically on Louisiana Medicare. The state has a relatively high chronic disease burden, an older average population, and low levels of health and digital literacy [51,52]. These are all factors that can slow early adoption and increase initial contact with providers as patients and clinicians adapt to new tools. At the same time, Louisiana’s high poverty rate and large rural population may limit broadband access [53], contributing to declining utilization after initial implementation phases stabilize. Finally, reimbursement for RPM under other payers (Medicaid and commercial programs) was only gradually expanded in Louisiana after 2019 as well, constraining early uptake and shaping provider behavior during the study period. These contextual features suggest that both the learning period and structural factors unique to Louisiana likely influenced the temporal dynamics observed in this study.

From a policy and clinical perspective, these findings have dual and uncertain implications. While increased short-term utilization may raise costs, if the increased visits reflect timely, preventive, or necessary care, the investment could yield downstream savings through decreased complications and their associated treatments. Conversely, if the increased encounters are duplicative or not clinically necessary, they could add costs. Policymakers and payers should therefore interpret early utilization spikes with caution. To prove that these visits should be encouraged, future research should further assess costs and clinical endpoints, which can clarify whether observed utilization patterns translate into value-based outcomes.

In the meantime, these results do offer some recommendations on how to maximize the benefits of RPM and potential challenges that need to be mitigated. First, providers should prioritize patient education initiatives; ensure that patients, especially older adults in Medicare, receive training on how to collect, interpret, and respond to RPM data. This finding is supported by a recent systematic review showing that RPM was more effective when patients had education and support in interpreting data [22]. Education programs can help enhance patient engagement and self-management while preventing the misuse of health care resources. Additionally, health care providers need structured support, including training on integrating RPM into care delivery and systems in place, to ensure that they have the resources to analyze RPM data.

### Limitations

As this study is observational in nature, the ability to assess causality is limited due to the potential for confounding and the lack of randomization. There is an inherent bias in how patients receive RPM. While the methodology attempts to overcome this, it may not have been able to completely eliminate biases in patient selection, which could bias the results away from the null because RPM patients are already engaging with the health system. Similarly, we handled confounding through IPTW and using controls, but some confounding may still be present. Additionally, the findings have limited generalizability as the population with diabetes and hypertension in Louisiana Medicare may differ from other populations. Therefore, these results may not be applicable when assessing or developing policy for other settings, including younger patients, other insurance types, or in other states.

Similarly, this study focused only on RPM through 2020, which was before the large increase in RPM and telehealth utilization due to COVID-19, and the study results may not be the same as those reported today, as some of the barriers mentioned here are less of a concern, resulting in results biased towards the null.

Moreover, this study did not assess the duration of RPM utilization or the number of times a physician checked the RPM data. Claims data do not allow assessment of patient engagement with RPM, and this study only looks at RPM prescription, not true utilization, as an outcome, although actual use mitigates the impact. Therefore, this might bias results towards the null if this group is not engaging with the technology or bias upwards if this population is engaged with RPM technology.

Similarly, the RPM CPT code does not indicate the type of device a patient is using, and as a result, these findings cannot be applied to certain RPM types, such as continuous glucose monitoring, and represent the larger impact of RPM as a treatment option. Finally, a significant limitation of this study is that it did not assess the appropriateness of utilization or the impact on clinical outcomes. If the increase in utilization stems from patients receiving timely care or appropriate care, health outcomes may improve.

This study is innovative in its use of comprehensive real-world evidence from statewide Medicare claims to evaluate RPM among older adults with diabetes or hypertension, a population underrepresented in prior studies. The analysis distinguishes short-term onboarding effects from longer-term stabilization in health care use, expanding on potential reasons for inconsistent findings in earlier research. Conducted during a period of expanding RPM reimbursement, these results provide timely insights for clinicians and payers on targeting, education, and workflow integration to optimize RPM value and reduce low-value encounters.

### Conclusions

This study assessed the association between RPM and HCRU among Louisiana Medicare beneficiaries with diabetes or hypertension. We found that RPM was associated with a modest but statistically significant increase in health care utilization, highlighting important questions about how RPM is implemented and integrated into routine care. This study contributes to the existing literature by examining RPM at scale in a real-world Medicare population using administrative claims data, rather than focusing on short-term clinical outcomes or small pilot programs. Unlike prior studies that primarily evaluate changes in biometric measures, our findings underscore the need to consider utilization patterns as an early indicator of how RPM influences care delivery. Although the current analysis could not assess the clinical appropriateness or downstream value of these encounters, the results suggest that initial increases in utilization may reflect enhanced monitoring, care coordination, or unmet need. Future work should link claims with clinical and cost data, examine patient engagement and digital literacy, and identify implementation strategies that ensure RPM supports high-value, equitable care delivery.

## Supplementary material

10.2196/80782 Multimedia Appendix 1 Residual Plots.

10.2196/80782 Multimedia Appendix 2 Sensitivity Analyses.

## Abbreviations

CITSA: controlled interrupted time series
COPD: chronic obstructive pulmonary disease
CPT: common procedure terminology
ER: emergency room
HCRU: health care resource utilization
ICD: International Classification of Diseases
IPTW: Inverse Probability Treatment Weight
RPM: remote patient monitoring
STROBE: Strengthening the Reporting of Observational Studies in Epidemiology
