# Supplementary material for: Remote Patient Monitoring in Louisiana Medicare Beneficiaries With Diabetes or Hypertension: Retrospective Cohort Study of 2016-2020 Claims Data
Source: J Med Internet Res. 2026 Mar 3;28:e80782. doi: 10.2196/80782 (PMC12979976; doi:10.2196/80782)
Supplement: Multimedia Appendix 2 [file jmir-v28-e80782-s002.docx]

Appendix 2: Robustness Tests

Table 1. Results Clustered by Zip code

| ​ | **All-Cause**  **Visits** | **Diabetes**  **Visits** | **Hypertension Visits** |
| --- | --- | --- | --- |
| **Outpatient Visits** | | | |
| **Post-Treatment Level** | 2.28***  (0.10) | 0.67***  (0.03) | 0.19***  (0.02) |
| **Post -Treatment Trend**​ | -0.04***  (0.002) | -0.01***  (0.00) | -0.003***  (0.00) |
| **Emergency Room Visits** | | | |
| **Post-Treatment Level** | 0.08***  (0.01) | 0.01***  (0.00) | 0.01***  (0.00) |
| **Post -Treatment Trend**​ | -0.001***  (0.00) | -0.0003***  (0.00) | -0.001***  (0.00) |
| **Inpatient Visits** | | | |
| **Post-Treatment Level** | 0.03***  (0.00) | 0.01***  (0.00) | 0.003**  (0.00) |
| **Post -Treatment Trend**​ | -0.0005***  (0.00) | -0.0001***  (0.00) | -0.0004** (0.00) |

 Note: All outcomes are at the monthly level per beneficiary

 Table 2. Only Beneficiaries with 12 Months Pre-Post AND only the 12 Months Pre-Post

| ​ | **All-Cause**  **Visits** | **Diabetes**  **Visits** | **Hypertension Visits** |
| --- | --- | --- | --- |
| **Outpatient Visits** | | | |
| **Post-Treatment Level** | 4.20***  (0.62) | 1.88***  (0.21) | 0.11  (0.17) |
| **Post -Treatment Trend**​ | -0.07***  (0.01) | -0.03***  (0.00) | -0.002  (0.00) |
| **Emergency Room Visits** | | | |
| **Post-Treatment Level** | 0.20***  (0.04) | 0.05*** (0.01) | 0.03*** (0.01) |
| **Post -Treatment Trend**​ | -0.003***  (0.00) | -0.001***  (0.00) | -0.0005 *** (0.00) |
| **Inpatient Visits** | | | |
| **Post-Treatment Level** | 0.05** (0.03) | 0.01  (0.01) | 0.012 (0.00) |
| **Post -Treatment Trend**​ | -0.001** (0.00) | -0.0003* (0.00) | -0.0002 (0.00) |

 Note: All outcomes are at the monthly level per beneficiary

 Table 3. Only Beneficiaries with 6 Months Pre-Post AND only the 6 Months Pre-Post

| ​ | **All-Cause**  **Visits** | **Diabetes**  **Visits** | **Hypertension Visits** |
| --- | --- | --- | --- |
| **Outpatient Visits** | | | |
| **Post-Treatment Level** | 4.19***  (1.06) | 2.16***  (0.29) | 0.25  (0.32) |
| **Post -Treatment Trend**​ | -0.07***  (0.53) | -0.04***  (0.00) | -0.004  (0.01) |
| **Emergency Room Visits** | | | |
| **Post-Treatment Level** | 0.30***  (0.01) | 0.10***  (0.00) | 0.03 (0.02) |
| **Post -Treatment Trend**​ | -0.004***  (0.00) | -0.002***  (0.00) | -0.0005  (0.00) |
| **Inpatient Visits** | | | |
| **Post-Treatment Level** | 0.07 (0.00) | 0.01  (0.00) | 0.02 (0.00) |
| **Post -Treatment Trend**​ | -0.001* (0.00) | -0.0002 (0.00) | -0.004  (0.00) |

Note: All outcomes are at the monthly level per beneficiary

Table 4. Exclude 2020 Due to Covid

| ​ | **All-Cause**  **Visits** | **Diabetes**  **Visits** | **Hypertension Visits** |
| --- | --- | --- | --- |
| **Outpatient Visits** | | | |
| **Post-Treatment Level** | 2.38***  (0.17) | 0.84***  (0.07) | 0.12***  (0.02) |
| **Post -Treatment Trend**​ | -0.04***  (0.001) | -0.01***  (0.00) | -0.002***  (0.00) |
| **Emergency Room Visits** | | | |
| **Post-Treatment Level** | 0.08***  (0.01) | 0.01***  (0.00) | 0.003  (0.00) |
| **Post -Treatment Trend**​ | -0.001***  (0.00) | -0.003***  (0.00) | -0.0001  (0.00) |
| **Inpatient Visits** | | | |
| **Pre-Treatment Trend**​ | 0.07***  (0.00) | 0.01***  (0.00) | 0.02***  (0.00) |
| **Post-Treatment Level** | -0.001***  (0.00) | -0.0002***  (0.00) | -0.0004***  (0.00) |

Note: All outcomes are at the monthly level

Table 5. Seasonality With Calendar Month

| ​ | **All-Cause**  **Visits** | **Diabetes**  **Visits** | **Hypertension Visits** |
| --- | --- | --- | --- |
| **Outpatient Visits** | | | |
| **Post-Treatment Level** | 2.28***  (0.01) | 0.67***  (0.001) | 0.19***  (0.001) |
| **Post -Treatment Trend**​ | -0.04***  (0.0001) | -0.01***  (0.00) | -0.003***  (0.00) |
| **Emergency Room Visits** | | | |
| **Post-Treatment Level** | 0.08***  (0.01) | 0.01***  (0.00) | 0.01*** (0.00) |
| **Post -Treatment Trend**​ | -0.001***  (0.00) | -0.0003***  (0.00) | -0.0001 *** (0.00) |
| **Inpatient Visits** | | | |
| **Pre-Treatment Trend**​ | 0.03***  (0.001) | 0.01***  (0.00) | 0.003*** (0.00) |
| **Post-Treatment Level** | -0.001***  (0.00) | -0.0001***  (0.00) | -0.0004***  (0.00) |

Note: All outcomes are at the monthly level

Table 6. Seasonality With Calendar Month of Treatment Control

| ​ | **All-Cause**  **Visits** | **Diabetes**  **Visits** | **Hypertension Visits** |
| --- | --- | --- | --- |
| **Outpatient Visits** | | | |
| **Post-Treatment Level** | 2.28***  (0.01) | 0.67***  (0.004) | 0.19***  (0.001) |
| **Post -Treatment Trend**​ | -0.04***  (0.0001) | -0.01***  (0.00) | -0.003***  (0.00) |
| **Emergency Room Visits** | | | |
| **Post-Treatment Level** | 0.08***  (0.01) | 0.01***  (0.00) | 0.01*** (0.00) |
| **Post -Treatment Trend**​ | -0.001***  (0.00) | -0.0003***  (0.00) | -0.0001***  (0.00) |
| **Inpatient Visits** | | | |
| **Pre-Treatment Trend**​ | 0.03***  (0.001) | 0.01***  (0.00) | 0.003*** (0.00) |
| **Post-Treatment Level** | -0.001***  (0.00) | -0.0001***  (0.00) | -0.0004***  (0.00) |

Note: All outcomes are at the monthly level
